# Performance and user-friendliness of the rapid antigen detection tests QuickVue Dipstick Strep A test and DIAQUICK Strep A Blue Dipstick for pharyngotonsillitis caused by *Streptococcus pyogenes* in primary health care

**DOI:** 10.1007/s10096-020-04034-z

**Published:** 2020-09-29

**Authors:** Una Ørvim Sølvik, Elisabet Eriksson Boija, Sara Ekvall, Afamia Jabbour, Anne Christin Breivik, Gunnar Nordin, Sverre Sandberg

**Affiliations:** 1 Department of Global Public Health and Primary Care, Faculty of Medicine, University of Bergen, Bergen, Norway; 2 Equalis AB, Uppsala, Sweden; 3 Department of Clinical Microbiology, Office for Medical Service, Region Skåne, Lund, Sweden; 4 Noklus (Norwegian Organization for Quality Improvement of Laboratory Examinations), Haraldsplass Deaconess Hospital, Bergen, Norway; 5 Department of Medical Biochemistry and Pharmacology, Haukeland University Hospital, Bergen, Norway

**Keywords:** Streptococcus pyogenes, Rapid antigen detection test, Diagnostic sensitivity, Diagnostic specificity, Primary health care, User-friendliness

## Abstract

Sensitivity and specificity of rapid antigen detection tests (RADTs) for detection of group A hemolytic streptococcus (GAS) vary. The purpose is to present the first SKUP (Scandinavian evaluation of laboratory equipment for point of care testing) evaluations concerning the assessment of the diagnostic performance and user-friendliness of two RADTs for detection of GAS when used under real-life conditions in primary health care. Throat samples were collected in duplicates at primary health care centers (PHCCs) from patients with symptoms of pharyngitis. The performance of QuickVue Dipstick Strep A test (307 samples) and DIAQUICK Strep A Blue Dipstick (348 samples) was evaluated using culture results at a clinical microbiology laboratory as comparison. The user-friendliness was evaluated using a questionnaire. The diagnostic sensitivity was 92% (90% confidence interval (CI) 87–96%) and 72% (90% CI 65–79%), while the diagnostic specificity was 86% (90% CI 81–90%) and 98% (90% CI 96–99%) for QuickVue Dipstick Strep A test and DIAQUICK Strep A Blue Dipstick, respectively. Both RADTs obtained acceptable assessments for user-friendliness and fulfilled SKUP’s quality goal for user-friendliness. The diagnostic sensitivity for QuickVue Dipstick Strep A test and the diagnostic specificity for DIAQUICK Strep A Blue Dipstick in this objective and supplier-independent evaluation were higher compared with previous meta-analyses of RADTs. However, the diagnostic specificity for QuickVue Dipstick Strep A test and the diagnostic sensitivity for DIAQUICK Strep A Blue Dipstick were lower compared with previous meta-analyses of RADTs.

## Introduction

Group A hemolytic streptococcus (*Streptococcus pyogenes*; *S. pyogenes*) (GAS) is the most frequent bacterial cause of infectious pharyngitis. GAS is estimated to account for 20 to 40% of cases of pharyngitis in children and 5 to 15% in adults [1, 2]. Common signs and symptoms of the disease include sore throat, fever, tonsillar exudates, and swollen cervical lymph nodes. Diagnosis based on clinical features alone is difficult because symptoms of bacterial pharyngitis overlap with those of viral pharyngitis. The Norwegian guidelines for primary health care on streptococcal pharyngitis recommend using rapid antigen detection tests (RADTs) for GAS in patients fulfilling at least two of the four Centor criteria (fever, anterior cervical lymphadenopathy, tonsillar rubor and exudates, and lack of cough) [3, 4]. In the Danish guidelines, two or more of the modified Centor criteria [5] must be fulfilled [6], while the Swedish guidelines recommend usage of RADTs upon clinical suspicion of bacterial pharyngitis and fulfillment of three or four Centor criteria [7]. Antibiotics are recommended if positive RADT [4, 6, 7].

There are numerous RADT methods [8], and sensitivity and specificity vary [8–11]. The most widespread and used RADTs in clinical practice are based on enzyme immunoassay (EIA) methodology, also known as immunochromatographic or lateral-flow assays. For these tests, the diagnostic sensitivity and specificity vary between 59 and 100% and between 54 and 100%, respectively [8, 11]. In addition to the method, several factors influence the performance of RADTs, such as duration of symptoms, disease severity, adequacy, and quality of the specimen, as well as the test operator [12–14]. Thus, it is important that the performance is evaluated by the intended user.

Scandinavian evaluation of laboratory equipment for point of care testing (SKUP) is a collaboration between the Scandinavian countries Sweden, Norway, and Denmark. The purpose of SKUP is to improve the quality of near-patient testing in Scandinavia by providing objective and supplier-independent information about analytical quality and user-friendliness of laboratory equipment [15]. SKUP generates this information by organizing evaluations which follow guidelines complied by SKUP. The analytical quality, diagnostic performance, and user-friendliness are assessed according to pre-set quality goals. The user-friendliness survey is an important satisfaction tool, which could help the users in choice of RADTs. Since no performance criteria for RADTs are specified in guidelines, the quality goal set by SKUP is based on previous results as well as expert opinions [16–18]. SKUP has evaluated 11 in vitro diagnostic RADTs for detection of GAS since 2003 [15]. The first nine focused on evaluation of analytical performance only and did not include evaluation of the diagnostic performance by the intended users. Reports from all SKUP evaluations are available on SKUPs homepage [15]. This paper presents the first SKUP evaluations concerning the assessment of the diagnostic accuracy performance and user-friendliness of two RADTs for GAS, QuickVue Dipstick Strep A test and DIAQUICK Strep A Blue Dipstick, when used under real-life conditions by the intended users in primary health care.

## Material and methods

In these prospective studies, data from two SKUP evaluations of RADTs for GAS were used. The tests were QuickVue Dipstick Strep A test (Quidel Corporation, San Diego, California, USA) (SKUP/2015/107) [15] and DIAQUICK Strep A Blue Dipstick (DIALAB GmbH, Neudorf, Austria) (SKUP/2018/114) [15] (referred to as QuickVue and DIAQUICK, respectively, throughout this paper), and the evaluations were performed in 2015 and 2018, respectively [15]. An ethical approval for the evaluations was not necessary because the evaluations were considered as quality assurance projects. The Standards for Reporting Diagnostic Accuracy (STARD) list [19] has been followed.

### Recruitment of patients

Consecutive patients with severe symptoms of pharyngitis seeking care in primary health care centers (PHCCs) in Skåne county (Sweden) were asked if they were willing to participate in the study. In this region, there is a low risk of serious complications caused by GAS [7]. In the evaluation of QuickVue, seven PHCCs recruited patients from February to March 2015, and in the evaluation of DIAQUICK, four PHCCs recruited patients from February to April 2018. Participation was voluntary and verbal consent was considered to be sufficient (for patients < 18 years, a parent also needed to consent). The patients were included by the Centor criteria [3] where the patient is assessed on four criteria, with one point added for each positive criterion: (1) history of fever (> 38.0 °C), (2) tonsillar exudates, (3) tender anterior cervical adenopathy, and (4) absence of cough. Individuals with pharyngitis suspected to be bacterial by the physician or nurse and fulfilled at least two of the Centor criteria were included in the study. This is according to the Norwegian guidelines [4]. Exclusion criteria were antibiotic treatment during the last 14 days, due to the risk of false-positive results.

### Handling of the samples and measurements with the RADTs

QuickVue and DIAQUICK are lateral-flow immunoassays detecting antigen from both viable (true positive) and nonviable (false positive) organisms of *S. pyogenes* directly from human throat swabs or culture colonies within 5 min. Assistant nurses and biomedical laboratory scientists (BLSs) in the participating PHCCs were trained to use the tests correctly by a local retailer. The training reflected the training normally given by the local retailer to customers. The clinical information was available to the performers of the RADT. Throat samples were collected in duplicates by rolling two swabs over the tonsils simultaneously: one swab for the measurement with the RADT and the other for the comparison method. The swabs were rubbed together to ensure equal distribution of sample material before running the tests. One swab was processed as described in the kit insert for the RADTs [20, 21]. Three different lots of each RADT were used for the purpose of having an evaluation less sensitive to the risk of a poor or good batch, but separate lot calculations were not performed. Reading of the strips was most times performed after 5 min. Strongly positive results were read after 1–4 min. For QuickVue, one was read after 10 min, and six were read after 6 min. The other swab intended for the comparison method was swirled in a tube with transport medium and kept in a refrigerator (2–8 °C) until it was sent in a cold box (8–14 °C) to the clinical microbiology laboratory the same day or for a few samples the following day.

### Internal and external analytical quality control for the RADTs

Internal analytical quality control samples, one negative and one positive, were included in the test kits for the RADTs. The producer of the RADTs assigned target values. Control samples were analyzed every day patient samples were analyzed. In the evaluation of DIAQUICK, the PHCCs alternated between a positive and a negative control. In addition, the built-in control features, such as the appearance of a control line, were examined for each RADT.

In both evaluations, each PHCC participated in one round of external quality assessment (EQA) from External Quality Assurance in Laboratory Medicine in Sweden (Equalis) which consisted of three control materials with different concentrations of antigen from non-viable Strep A bacteria. The target values were assigned by the producer of the control materials (a clinical microbiology laboratory in Sweden).

### User-friendliness of the RADTs

To evaluate the user-friendliness of the RADTs, a questionnaire divided into four sections was used: (1) rating of the ease of usage of the rapid test, (2) rating of the information in the kit insert, (3) rating of time factors for the preparation and the measurement including stability of the tests and internal quality controls, and (4) rating of performing internal and external analytical quality controls.

The assistant nurses and BLSs at the PHCCs that used the RADTs filled in sections 1 and 2 (Tables 3 and 4) at the end of the evaluation period. SKUP filled in sections 3 and 4 (Tables 5 and 6) in addition to topics marked with gray color for sections 1 and 2. For each question, there were three given ratings: unsatisfactory, intermediate, and satisfactory. To achieve the overall rating satisfactory, the total rating of satisfactory in all four sections had to be reached.

### The comparison method

In the absence of a reference measurement method, culturing of *S. pyogenes* in a clinical microbiology laboratory (Department of Clinical Microbiology, Office for Medical Service, Region Skåne in Lund, Sweden), was used as comparison method. The laboratory is accredited by the Swedish Board for Accreditation and Conformity Assessment (Swedac) for culturing of beta hemolytic groups A, C, and G streptococci. The RADT results were available to the assessors of the comparison method. With a few exceptions, the culturing started the same day as the sample collection. If the swab arrived late afternoon, the samples were kept refrigerated overnight before culturing. The patient samples were cultured once. The samples (30 μL of E-swab-liquid) were spread on double-layered sheep-blood agar plates (Columbia II agar, BD) and incubated in an anaerobe environment at 37 °C for 16–20 h. In case of growth of typical colonies with beta hemolysis, the streptococci were characterized with an agglutination test (Streptex™, Thermo Scientific, Oslo, Norway) to detect the Lancefield group antigens (A, B, C, G). The results of RADT were reported to the laboratory as negative, positive, or invalid and were ready within 24 h for beta hemolytic streptococci. The performers of the RADTs were usually informed if the comparison method (culture) results deviated from the rapid test result.

The trueness of the comparison method (culture) was evaluated with EQA results. The EQA samples were provided by United Kingdom National External Quality Assessment Service (UK NEQAS). The clinical microbiology laboratory in Lund showed satisfactory results for culturing of beta hemolytic streptococci during the evaluation periods (2014–2015 for QuickVue and 2018 for DIAQUICK ).

When new batches of agar plates were prepared, an internal analytical quality control was performed by culture of the reference strain beta hemolytic group A CCUG 25571, on some of the new plates.

### Statistics and performance specifications

Calculations and statistical analysis were performed with Microsoft Excel. The intended sample size was estimated from the presumption of at least 100 true positive results from consecutively included patients in order to obtain acceptable confidence interval (CI) for diagnostic sensitivity and specificity for the RADTs. Based on previous evaluations performed in autumn, winter, or early spring, the prevalence of GAS was estimated to about 25% in the population tested for GAS. Thus, the goal was to recruit about 400 patients. The results of the RADTs achieved in the PHCCs were evaluated against the results of the culturing of samples from the same patients in the clinical microbiology laboratory (comparison method). If results were missing from the comparison method, the samples were excluded from the calculations.

The diagnostic sensitivity and specificity of the RADTs were calculated by comparing the test results in the PHCCs with the results from the clinical laboratory. Estimation of CI for binomial proportions was performed according to Adjusted Wald [22].

The performance specifications for diagnostic accuracy set by SKUP were diagnostic sensitivity > 80% and diagnostic specificity > 95%. The fraction of technical errors should be ≤ 2%.

## Results

### Patient samples

**Fig. 1:**
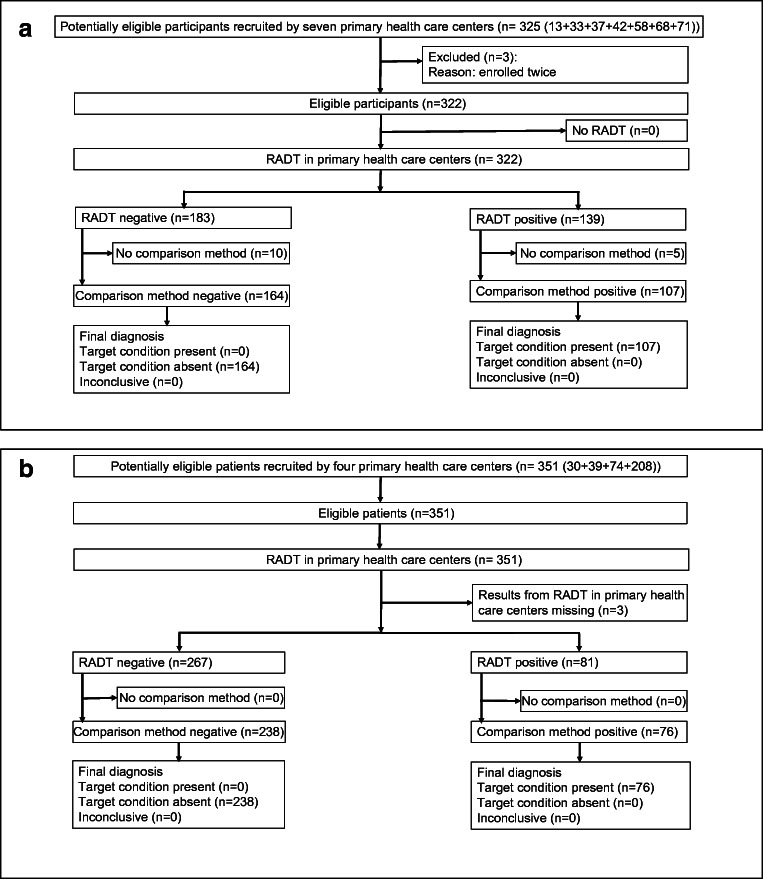
A modified Standards for Reporting Diagnostic Accuracy (STARD) diagram [19] showing the flow of participants in the evaluations of **a** QuickVue Dipstick Strep A test (performed in 2015) and **b** DIAQUICK Strep A Blue Dipstick (performed in 2018). No RADTs were inconclusive. The 42 (13%) patients with missing information about Centor criteria in the evaluation of QuickVue are included. RADT, rapid antigen detection test; Comparison method, culturing of Streptococcus pyogenes in a clinical microbiology laboratory

In total 322 and 351 patients provided samples both for the RADT method and for the comparison method in the evaluation of QuickVue and DIAQUICK, respectively (Table 1, Fig. 1 a and b). The collection of the throat samples was mainly reported as easy with no adverse events. Characteristics of the patients are given in Table 1. In the evaluation of QuickVue, 98 patients (30%) fulfilled two Centor criteria [3], 180 (56%) fulfilled three or four, and for 42 (13%) patients, there were missing information about Centor criteria. In the evaluation of DIAQUICK, 142 patients (41%) fulfilled two Centor criteria, and the remaining (209, 59%) fulfilled three or four. There were no indeterminate RADT or comparison method results. Culture results of 15 of the patient samples were missing in the evaluation of QuickVue, and three RADT results were missing in the evaluation of DIAQUICK. Thus, 307 samples were included in the calculations for QuickVue, and 348 samples were included in the calculations for DIAQUICK (Fig. 1 a and b). For patients with negative RADTs and culture results, there was no information about alternative diagnosis. There were no technical errors reported for the two RADTs.

**Table 1:** Characteristics of the patients that participated in the evaluations of QuickVue Dipstick Strep A test (performed in 2015) and DIAQUICK Strep A Blue Dipstick (performed in 2018) (SKUP/2015/107, SKUP/2018/114 [15])

	QuickVue Dipstick Strep A test	DIAQUICK Strep A Blue Dipstick
	*n* = 317	*n* = 351
Female, *n* (%)	183 (57)	197 (56)
Male, *n* (%)	134 (43)	154 (44)
Median age, year (range)	17 (0.67–86)	18 (0.67–88)
<10 years, *n* (%)	106 (33%)	129 (37%)

### Internal and external analytical quality control for the rapid antigen detection tests

In both evaluations, all measurements of both the positive and negative internal controls showed correct result (*n* = 146 QuickVue; *n* = 142 DIAQUICK). In the EQA, the PHCCs achieved correct results with the RADTs on all three samples.

### Diagnostic sensitivity and specificity

The number of true positive and negative, and false positive and negative results, as well as diagnostic sensitivity and specificity for QuickVue and DIAQUICK, are shown in Table 2. The 42 patients with missing information about Centor criteria were included in the calculations. Exclusion of these results did not change the results (data not shown). For DIAQUICK, 16 of the 29 false negative results displayed sparse growth of colonies; eight displayed moderate growth of colonies. For QuickVue, there was no connection between the number of colonies and the false negative results; sparse, moderate, and abundant growths were among the nine false negative results. For the samples read after 10 and 6 min, there were both positive and negative results and no weak positive. One of the four throat samples giving a group C hemolytic streptococcus (Strep C) positive culture showed a positive result with QuickVue. All other Strep C (*n* = 4) or group G hemolytic streptococcus (Strep G) (*n* = 11) positive cultures tested negative with QuickVue. The sample giving a Strep C-positive culture and a positive result with QuickVue could indicate interference; however, the data set was too small to draw any final conclusions. Furthermore, the sample with the Strep C positive culture showed negative real-time PCR result [15], which confirms the culturing being negative for Strep A. Several of the false positive results also showed negative results with real-time PCR [15], which indicates that the QuickVue test showing positive result for the strep C-positive patient was just a random error. In the evaluation of DIAQUICK, there were 10 Strep C- and 7 Strep G-positive cultures in addition to the 105 Strep A-positive cultures. All the 17 Strep C- and G-positive cultures were negative on DIAQUICK. Thus, none of the false positive results on DIAQUICK were due to Strep C or Strep G. QuickVue fulfilled the quality goals set by SKUP for diagnostic sensitivity, and DIAQUICK fulfilled the quality goals for diagnostic specificity.

**Table 2:** Number of true positive and negative, and false positive and negative results, diagnostic sensitivity and specificity (including 90% CI), for QuickVue Dipstick Strep A test (evaluation performed in 2015) and DIAQUICK Strep A Blue Dipstick (evaluation performed in 2018) (SKUP/2015/107, SKUP/2018/114) [15]

	QuickVue Dipstick Strep A test	DIAQUICK Strep A Blue Dipstick
True positive results, *n*	107	76
False negative results, *n*	9	29
True negative results, *n*	164	238
False positive results, *n*	27	5
Diagnostic sensitivity, % (90% CI)	92% (87–96%)	72% (65–79%)
Diagnostic specificity, % (90% CI)	86% (81–90%)	98% (96–99%)

*CI* confidence interval

### Rating of user-friendliness of the RADTs

All four sections in the questionnaire about user-friendliness for both QuickVue and DIAQUICK were rated as satisfactory (Tables 3, 4, 5, and 6). However, there were three intermediate ratings by the PHCCs for the ease of usage of the rapid test for QuickVue and four for DIAQUICK (footnotes in Table 3). For the information in the kit insert, there were two intermediate and one unsatisfactory assessment for QuickVue (footnotes in Table 4). In addition, there were some positive comments (Table 4).

**Table 3:** Rating of the ease of usage of the rapid tests QuickVue Dipstick Strep A Test (evaluation performed in 2015) and DIAQUICK Strep A Blue Dipstick (evaluation performed in 2018) by the primary health care personnel and the SKUP coordinator. Comments for intermediate or non-satisfactory assessments, provided by the assistant nurses and BLSs at the PHCCs that used the RADTs or SKUP, are given in footnotes (SKUP/2015/107, SKUP/2018/114) [15]

Topic	Rating	
	QuickVue Dipstick Strep A Test	DIAQUICK Strep A Blue Dipstick
	(n=7 PHCCs)	(n=4 PHCCs)
To prepare the test / instrument	Satisfactory^a^	Satisfactory^h^
To prepare the sample	Satisfactory^a^	Satisfactory^h^
Application of specimen	Satisfactory	Satisfactory
Number of procedure step	Satisfactory	Satisfactory
Instrument / test design	Intermediate^b,c^	Satisfactory^h^
Reading of the test result	Easy	Easy^i^
Sources of errors	Satisfactory	Satisfactory
Hygiene, when using the test	Satisfactory	Satisfactory
Size and weight of package	Satisfactory	Satisfactory
**Storage conditions for tests, unopened package**	**Satisfactory**^d^	**Satisfactory**^j^
**Storage conditions for tests, opened package**	**Satisfactory**^d^	**Satisfactory**^j^
**Environmental aspects: waste handling**^e^	**Satisfactory**^f^	**Satisfactory**^k^
**Intended users**^g^	**Satisfactory**	**Satisfactory**
Total rating by SKUP	Satisfactory	Satisfactory

^a^It was difficult to get the right amount of reagent, often too much (when squeezing the bottles by mistake). One intermediate assessment for each of the two topics

^b^The test rack in paper was unstable, would have liked racks in plastic instead. One intermediate assessment

^c^The disposable packages with the dipsticks were difficult to open. One unsatisfactory assessment

^d^+ 15 °C to + 30 °C

^e^Viable bacteria always have to be handled with special precautions.

^f^Sorted waste

^g^Health care personnel or patients

^h^The diameter of the extraction tube is too small, making it difficult to put drops and swab into it. Difficult to take out the single-packed test sticks. One intermediate assessment for each of the three topics

^i^A bit too weak lines sometimes. One intermediate assessment

^j^+ 2 °C to + 30 °C (SKUP rates + 15 °C to + 30 °C as satisfactory)

^k^Special precautions

Positive comment for QuickVue Dipstick Strep A test: Easy to work with, even for inexperienced personnel. Positive comments for DIAQUICK Strep A Blue Dipstick: Satisfactory test functions, good size package.

Topics marked in bold were rated by SKUP

*BLS* biomedical laboratory scientist, *PHCCs* primary health care centers, *RADT* rapid antigen detection test, *SKUP* Scandinavian evaluation of laboratory equipment for point of care testing

**Table 4:** Rating of the information in the kit insert for the rapid tests QuickVue Dipstick Strep A test (evaluation performed in 2015) and DIAQUICK Strep A Blue Dipstick (evaluation performed in 2018) by the primary health care personnel and the SKUP coordinator. Comments for intermediate or non-satisfactory assessments, provided by the assistant nurses and BLSs at the PHCCs that used the RADTs or SKUP, are given in footnotes (SKUP/2015/107, SKUP/2018/114) [15]

Topic	Rating	
	QuickVue Dipstick Strep A Test	DIAQUICK Strep A Blue Dipstick
	*(n=7 PHCCs)*	*(n=4 PHCCs)*
General impression	Satisfactory^a^	Satisfactory
Preparations / Pre-analytic procedure	Satisfactory	Satisfactory
Specimen collection	Satisfactory^b^	Satisfactory
Measurement procedure	Satisfactory	Satisfactory
Reading of result	Satisfactory	Satisfactory
Description of the sources of error	Satisfactory	Satisfactory
Help for troubleshooting	Satisfactory^c^	Satisfactory
Readability / Clarity of presentation	Satisfactory	Satisfactory
**Measurement principle**	**Satisfactory**	**Satisfactory**
**Available insert in Danish, Norwegian, Swedish**	**Satisfactory**	**Intermediate**^d^
Total rating by SKUP	Satisfactory	Satisfactory

^a^A bit difficult to evaluate a short insert (2 pages), but do not miss any information beside the ones given below. One intermediate assessment

^b^There were no illustrations for specimen collection. One unsatisfactory assessment

^c^Would like the contact information to be more visible, and also an e-mail address to be included. One intermediate assessment

^d^Not available in Danish

Positive comments for QuickVue Dipstick Strep A test: easy to understand, good illustrations, simple language, and laminated quick guide. No technical errors or failed measurements were reported.

Topics marked in bold were rated by SKUP

*BLS* biomedical laboratory scientist, *PHCCs* primary health care centers, *RADT* rapid antigen detection test, *SKUP* Scandinavian evaluation of laboratory equipment for point of care testing

**Table 5:** Rating of time factors for the preparation and the measurement including stability of the rapid tests QuickVue Dipstick Strep A test (evaluation performed in 2015) and DIAQUICK Strep A Blue Dipstick (evaluation performed in 2018) and internal quality controls by the SKUP coordinator (SKUP/2015/107, SKUP/2018/114) [15]

Topic	Time		Rating of both RADTs
	QuickVue Dipstick Strep A test	DIAQUICK Strep A Blue Dipstick	
Required training time	< 2 h	<2 h	Satisfactory
Durations of preparations/pre-analytical time	< 6 min	<6 min	Satisfactory
Duration of analysis	< 10 min	< 10 min	Satisfactory
Stability of test, unopened package	> 5 months	>5 months	Satisfactory
Stability of test, opened package^a^	> 30 days	> 30 day or disposable	Satisfactory
Stability of quality control material, unopened	> 5 months	> 5 months	Satisfactory
Stability of quality control material, opened	> 6 days or disposable	> 6 days or disposable	Satisfactory
Total rating by SKUP			Satisfactory

^a^The stability of the reagent solutions does not change when opened. Dipsticks are individually packed and opened right before use

*RADT* rapid antigen detection test, *SKUP* Scandinavian evaluation of laboratory equipment for point of care testing

**Table 6:** Rating of performing internal and external analytical quality control of the rapid tests QuickVue Dipstick Strep A test (evaluation performed in 2015) and DIAQUICK Strep A Blue Dipstick (evaluation performed in 2018) by the SKUP coordinator (SKUP/2015/107, SKUP/2018/114) [15]

Topic	Rating	
	QuickVue Dipstick Strep A test	DIAQUICK Strep A Blue Dipstick
Reading of the internal quality control ^a^	Satisfactory	Satisfactory
Usefulness of the internal quality control	Satisfactory	Satisfactory
External quality control	Satisfactory	Satisfactory
Total rating by SKUP	Satisfactory	Satisfactory

^a^In addition to the positive and negative controls included in the kit, several procedural control steps are built into the test

*RADT* rapid antigen detection test, *SKUP* Scandinavian evaluation of laboratory equipment for point of care testing

## Discussion

In these studies, the diagnostic performance and user-friendliness of two RADTs for GAS were assessed by the intended user under real-life conditions in primary health care. The diagnostic sensitivity was 92% (95% CI 87–96%) and 72% (95% CI 65–79%), while the diagnostic specificity was 86% (95% CI 81–90%) and 98% (95% CI 96–99%) for QuickVue and DIAQUICK, respectively. Both QuickVue and DIAQUICK obtained acceptable assessment for user-friendliness and thus fulfilled SKUPs quality goal. Very few studies have previously reported results for user-friendliness for RADTs [23].

For QuickVue, the diagnostic sensitivity in the present study was higher than pooled estimates obtained from meta-analysis for EIA (85% (95% CI 83–88%) for children [8] and 86% (95% CI 81–91%) for adults [10]), and fulfilled SKUPs quality goal for diagnostic sensitivity (> 80%). The diagnostic specificity for QuickVue, however, is lower than the pooled specificity in meta-analysis for EIA for children (96%, 95% CI 95–97%) and for adults (97%, 95% CI 96–99%) [10]) and from that reported by the manufacturer of the test (94%, 95% CI 91–97%) [20]. QuickVue did not fulfill the quality goal set by SKUP for diagnostic specificity (> 95%). However, the specificity of QuickVue in our study was within the range of reported specificity for EIA (54–100%) [8, 11]. In our evaluation of QuickVue, 18 of the 27 (66%) false positive results were from two sites representing 39% (121/309) of all tests. The remaining five sites accounted for 33% (9/27) of the false positive, representing 61% of the samples, giving a diagnostic specificity of 92%. In addition, of the 27 false positive results, five (18%) were reported as weakly positive (weak test line) by the PHCCs. Thus, user specific performance may partly explain the low specificity obtained for QuickVue in our study. Time to culture could also lead to “false positive” results since GAS does not survive at 4 °C [24] and may affect the result of the culture. Anyhow, in the evaluations, the standard procedure for sending throat samples to a laboratory for culturing used in primary health care was followed.

For DIAQUICK, the diagnostic specificity in the present study was higher than pooled estimates from meta-analysis for EIA for children (96%, 95% CI 95–97%) [8] and for adults (97%, 95% CI 96–99%) [10]), and fulfilled SKUPs quality goal for diagnostic specificity (> 95%). The five false positive results for DIAQUICK were from one site, but we have no information whether these were weakly positive or not. The site accounted for about 60% of the samples (208/351) representing 39% (121/309) of all tests.

The diagnostic sensitivity for DIAQUICK in our study, however, is lower than the pooled sensitivity in meta-analysis for EIA in children (85%, 95% CI 83–88%) [8] and adults (86%, 95% CI 81–91%) [10]), and did not fulfill SKUPs quality goal for diagnostic sensitivity (> 80%). The diagnostic sensitivity is also lower than reported by the manufacturer of the test (97%, 95% CI 91–99%) [21], and in a prospective study from 2008 that was performed in an outpatient setting with children (95.8%) [25]. However, that study had several limitations including unclear description of the comparison method, and they used an older version of the DIAQUICK. Anyway, the diagnostic sensitivity of DIAQUICK in our study was within the range of reported sensitivity for EIA (59–96%) [9]. Since the evaluations were performed in a region with low risk of serious complications caused by GAS, a low sensitivity is a lesser problem.

The laboratory staff were informed about the results of the RADTs. This may have introduced the opportunity for bias in the examination and interpretation of the cultures, which could have a slight impact on the figures for sensitivity and specificity.

There is no global consensus for the quality goal for diagnostic sensitivity and specificity for RADTs since the importance of these parameters varies in different parts of the world. This might contribute to the wide range of diagnostic sensitivity for different RADTs. In high-income countries the risk of serious complications caused by GAS is low and healthcare focus on minimizing inappropriate use of antibiotics. High sensitivity might result in detection of GAS carriers and unnecessary treatment. Thus, high diagnostic specificity is more important than high sensitivity. The performance criteria set by SKUP for RADTs are based on previous studies performed in Scandinavian countries [16–18], and are also in line with the pooled estimates for diagnostic sensitivity and specificity from meta-analysis [9, 10]. Nevertheless, in the meta-analyses, there was great heterogeneity with a high variability in methodology for the included studies, and the authors disclose that they do not have strong confidence in the estimates due to high heterogeneity of the included studies [9, 10].

Some studies indicate that the sensitivity of RADTs vary with the spectrum of disease [12, 26–28], and an increased number of modified Centor criteria [5] has been shown to be associated with increased RADT sensitivity [12, 28]. However, in a meta-regression, there were no significant associations between clinical severity (assessed by modified Centor criteria [5]) and sensitivity and/or specificity of the RADTs [8]. In our study, the same percentage of the patients (almost 60%) had three or four Centor criteria in the evaluation of both QuickVue and DIAQUICK. QuickVue did fulfill SKUPs criteria for diagnostic sensitivity, and the diagnostic sensitivity did not change after exclusion of patients with only two Centor criteria (data not shown). Thus, the low sensitivity for DIAQUICK can probably not be explained by that a relatively high proportion of the patients had only two Centor criteria.

There is also a risk of false negative results from culturing and RADTs if the amount of secretion obtained from the throat samples is too small, and studies have shown that the sensitivity of RADTs increased considerable with inoculum size [13, 14]. In our evaluation of DIAQUICK, more than 83% of the false negative results displayed sparse or moderate growth of colonies which may indicate small amount of secret in the samples and may, thus, contribute to the low sensitivity. For culturing, there is additional risk for obtaining upper respiratory tract normal flora when collecting the throat samples resulting in false negative results. Culture performance is also affected by the conditions used for plating and incubation of the cultures [29], and there is no consensus on the details in the methods for culture of *S. pyogenes*. In our study, the cultures were performed in an accredited laboratory, and all results from the internal and external analytical quality control were satisfactory. Anyway, if the comparison method is either relatively insensitive or too sensitive, the performance of RADT may be evaluated erroneously. Neither the RADTs nor culturing, or any other methods, are able to distinguish between patients who are carriers of GAS and those who are actually infected with GAS.

In our study, about one third of the patients were < 10 years of age. However, this did probably not affect the results since meta-analysis found that the sensitivity and specificity of the RADTs when analyzed in pediatric studies alone were similar to the overall estimates [9]. Furthermore, the pooled sensitivity and specificity found in children and in mixed population of children and adults are very similar [11].

The prevalence of GAS in the tested population affects the performance of RADTs. In our studies, the prevalence of GAS was 38% in the evaluation of QuickVue and 30% in the evaluation of DIAQUICK. Used in a population with similar prevalence of disease, the positive predictive value (PPV) and the negative predictive value (NPV) for QuickVue is 80% and 95%, respectively. The corresponding numbers for DIAQUICK are 94% and 89%, respectively. In a population with a prevalence of 25% for GAS, the PPV and NPV would have been 69% and 97%, respectively for QuickVue. For DIAQUICK, the corresponding numbers are 92% and 41%, respectively.

The difference of prevalence of GAS in the same population in 2015 and 2018 is within the expected variation between seasons. We have no indication that the prevalence has been affected by genetic changes leading to altered virulence of *Streptococcus pyogenes* and prevalence of GAS infections [30].

In conclusion, the diagnostic sensitivities were 92% and 72%, and the diagnostic specificities were 86% and 98% for QuickVue and DIAQUICK, respectively in primary health care. Both RADTs obtained acceptable assessments for user-friendliness and fulfilled SKUPs quality goal for user-friendliness. There are several factors that can affect the performance of RADTs, and these studies provide an objective and supplier-independent information about analytical quality and user-friendliness when used under real-life conditions by the intended users.

## Data Availability

The data can be obtained by contacting SKUP.

## Abbreviations

BLS: Biomedical laboratory scientist
DIAQUICK: DIAQUICK Strep A Blue Dipstick
EQA: External quality assessment
NPV: Negative predictive value
PHCC: Primary health care center
PPV: Positive predictive value
Equalis: External Quality Assessment in Laboratory Medicine in Sweden
Noklus: Norwegian Organization for Quality Improvement of Laboratory Examinations
QuickVue: QuickVue Dipstick Strep A test
RADT: Rapid antigen detection test
SKUP: Scandinavian evaluation of laboratory equipment for point of care testing
*S. pyogenes*: *Streptococcus pyogenes*
Strep A: *Streptococcus pyogenes* group A
Strep C: Group C hemolytic streptococcus
Strep G: Group G haemolytic streptococcus
STARD: Standards for Reporting Diagnostic Accuracy
Swedac: Swedish Board for Accreditation and Conformity Assessment
UK NEQAS: United Kingdom National External Quality Assessment Service
